# Trap Parameters for the Fast OSL Signal Component Obtained through Analytical Separation for Various Quartz Samples

**DOI:** 10.3390/ma15238682

**Published:** 2022-12-06

**Authors:** Magdalena Biernacka, Alida Timar-Gabor, Zuzanna Kabacińska, Piotr Palczewski, Alicja Chruścińska

**Affiliations:** 1 Institute of Physics, Faculty of Physics, Astronomy and Informatics, Nicolaus Copernicus University in Toruń, ul. Grudziądzka 5, 87-100 Toruń, Poland; 2 Faculty of Environmental Science and Engineering, Babeş-Bolyai University, Fântânele 30, 400294 Cluj-Napoca, Romania; 3 Interdisciplinary Research Institute on Bio-Nano-Sciences, Babeş-Bolyai University, Treboniu Laurian 42, 400271 Cluj-Napoca, Romania

**Keywords:** traps, thermal stability, optically stimulated luminescence, quartz

## Abstract

Trap stability is essential in luminescence dating and thermochronometry. Trap depth and frequency factors determining the stability of the fast component of optically stimulated luminescence (OSL) in quartz, which is the most important in dating, have yet to be uniquely determined, especially for samples with an OSL signal not dominated by this component. One can determine them in OSL thermal depletion curve (OTDC) experiments. The separation of the fast OSL signal undisturbed by other OSL components is vital for obtaining accurate parameters for the traps of interest. This work presents a method of simultaneous thermal and optical stimulation using red light (620 nm) to separate the fast OSL component (the thermally modulated OSL method—TM-OSL). The OTDC experiment with the TM-OSL stimulation was used for the trap parameter determination on a variety of quartz samples, leading us to report for the first time, the trap parameters for the fast OSL component analytically separated in quartz from rock samples. The results obtained for these samples with the fast component of low intensity are consistent with those with an intensive fast OSL component. Results of OTDC measurements for all investigated quartz samples were tested for a wide range of irradiation doses.

## 1. Introduction

In this study, we work towards an accurate determination of the values of trap parameters (trap depth, denoted by *E,* and frequency factor, denoted by *s*) obtained in optically stimulated luminescence (OSL) isothermal experiments for quartz of various origins. Knowledge about the stability of investigated traps in quartz is crucial for applying the OSL method in dating and thermochronometry.

OSL dating method using quartz has an effective dating range of 10^1^–10^6^ years (e.g., [1]) and low-temperature sensitivity of ~35–60 °C (e.g., [2]) which makes it especially well-suited for Quaternary geochronology. For quartz, it was identified that the OSL signal comprises at least three components called, depending on the rate of decay, “fast”, “medium” and “slow” [3,4]. The primary condition for the OSL dating of the sediment is resetting its OSL signal by sunlight before the sediment layer formation. The fastest decaying OSL component is recognized as the one that guarantees accurate age estimation. That made the fast OSL component an object of extensive studies (e.g., Refs. [5,6]). The continuous-wave OSL (CW-OSL) method using blue (470 nm) stimulation light is commonly used to investigate the thermal stability of OSL traps in quartz (e.g., [7]). In the CW-OSL method, optical stimulation is performed with constant stimulation energy at a constant temperature. During such stimulation, the OSL signal from various optically active traps is depopulated simultaneously and the observed OSL signal represents the overall existing OSL components in the sample. The OSL signal decay rate for a particular electron trap depends on its optical cross-section (OCS), which stays constant at fixed stimulation energy and temperature. Thus, for two kinds of traps with different OCSs, the ratio of OSL decay rates remains unchanged; therefore, the ability of the CW-OSL method to separate the signal related to various traps is limited. However, in many studies, the trap parameters obtained in this way for sedimentary quartz samples of various geological origins show variability in *E* and *s* values, for example, *E* = 1.65 ± 0.02 eV and s = 5.01·10^13^ s^−^^1^ [8], *E* = 1.41 ± 0.13 eV and *s* = 4.25·10^11^ s^−^^1^ [9], *E* =1.70 ± 0.01 eV and *s* = 7.76·10^13^ s^−^^1^ [10]. Besides the variability in the obtained parameters, deviation from first-order kinetics was reported for the thermal depletion curves (e.g., Refs. [10,11,12]). These observations may be explained by the different contributions of the OSL components in the depleted OSL signal. The charge carrier transitions between traps, such as thermal transfer, as well as the significant variation of OSL components contributions from sample to sample can negatively affect the accuracy of the obtained values. Therefore, the isolation of the fast OSL component from other components is of the utmost importance. Previous works [13,14,15] proposed thermally modulated OSL to achieve such a separation and showed that the fast OSL component in quartz could be measured using stimulation at 620 nm. The TM-OSL method uses the dependency of the OCS on the temperature to separate the signal from various types of traps more effectively. Differences between the OCSs of various traps are more significant if the light of a longer wavelength is used for stimulation, and the OCS increases more dynamically with temperature. The stimulation light effectively empties the traps with the highest OCS if a suitable wavelength is applied. Moreover, the rising temperature causes a continuous OCS increase and the time necessary to depopulate the investigated traps is shortened. This ensures that the traps, which are not intended to be bleached, are emptied to a negligible extent. In order to observe a pure OSL signal, the TM-OSL stimulation should be accomplished in the temperature range where the thermal activation of investigated traps is minimal.

Recently OSL thermal depletion curves (OTDC) were constructed using TM-OSL_620 nm_ instead of CW-OSL_470 nm_ for analyzing the thermal stability of traps responsible for the fast OSL component in sedimentary quartz. The trap parameters found in this way were compared with those obtained using other methods such as initial rise, variable heating rate and TL peak fitting [16].

While determining these values for sedimentary quartz is important as they partially dictate the age range of dating, the situation is much more complex when rock quartz samples are used for thermochronometry. Thermal stability analysis of the OSL signal is crucial for the evaluation of the “closure temperature” [2,17]. Using erroneous values for these parameters to calculate the lifetime of a charge carrier in a trap leads to a false reconstruction of the time-temperature history of rocks in thermochronometry. Moreover, in the case of rock quartz, the OSL signal usually has a much lower intensity than in sedimentary quartz and the contribution of the fast OSL component is significantly lower (e.g., [10]). Despite this, the common trap parameters generally used in thermochronometry applications (see e.g., Refs. [18,19]) are those obtained for sedimentary samples dominated by the fast OSL component, namely *E* = 1.59 eV and *s* = 10^12.9^ s^−^^1^ obtained on the WIDG8 sample by Murray and Wintle [20] or *E* = 1.59 eV and *s* = 2.8·10^12^ s^−^^1^ obtained on sample LW 94/1 by Spooner and Questiaux [21]. Keeping in mind the variability observed in sediments, one should be cautious when *E* and *s* values obtained for the fastest blue—OSL component in sedimentary quartz are applied to thermochronometry analysis for quartz from rocks. For practical application in this field, rock samples need to be characterized.

Here, the thermally modulated method (TM-OSL_620 nm_) where the optical stimulation is conducted during linear heating of the sample was applied for investigating the thermal stability of the OSL signal of quartz extracted from granites. The results are compared to those obtained on sedimentary quartz. The influence of measurement parameters such as (i) preheat temperature, or in other words, the initial occupation of traps involved in the isothermal process, and (ii) the impact of irradiation dose on the isothermal analysis results are thoroughly investigated. Based on these investigations we report for the first time the analytical separation of the fast OSL signal component in quartz extracted from plutonic rocks and present recommendations for improving the accuracy of determination of trap parameters for the defect responsible for this signal in such problematic samples.

## 2. Materials and Methods

### 2.1. Samples

The following four samples of sedimentary quartz (coarse grain) were investigated:

- 2 MV 570—dia. 63–90 μm, a loess sediment from Mircea Voda, Romania. A detailed description of the preparation of sample 2 MV 570 can be found elsewhere [22], with references therein (in this work subsample 2 MV 570A was used);
- QC (calibration quartz)—dia. 180–200 μm, aeolian sediments, Jutland, the Radiation Research Division of the Technical University of Denmark in Risø (Risø DTU/RRD). A detailed description of the preparation of the QC sample can be found elsewhere [23,24].
- MR—dia. 150–250 μm, from ~8 to 9 ka old deposits ‘Silver Sands of Morar’, reworked intensely by fluvial and marine processes. The preparation of sample MR is described in detail by Schmidt et al. [25].
- FB—dia. 150–250 μm, Oligocene coastal sand from the Fontainebleau Sand Formation, from the time of the last marine intrusion into the Paris Basin (the Stampian Sea) ~35 Ma ago. The preparation of sample FB is described in detail in Kreutzer et al. [26] (in this work batch FB, subsample FB3A was used);

Two investigated samples of igneous (rock) quartz:

- GC—Catalina granite, dia. 250–500 μm, Cenozoic Granite with an age of about 26 Ma, Tucson, southeast Arizona, USA [27,28].
- GO—Oracle granite, dia. 500–1000 μm, Proterozoic granite with a crystallization age of about 1.4 Ga, Tucson, southeast Arizona, USA [27,28].

Quartz has been extracted by crushing, light mineral fraction separation by Wilfley table followed by centrifugation in diluted Na_6_[H_2_W_12_O_40_] × H_2_O (2.62 g/cm^3^ and 2.75 g/cm^3^) and 40 % hydrofluoric acid etching for 60 min followed by rinsing with 10% HCl to remove any acid-soluble fluoride precipitates.

### 2.2. Instrumentation

The OTDC experiment using TM-OSL_620 nm_ protocol was carried out using equipment set up based on a Risø TL/OSL- DA-20 System equipped with an additional stimulation light source. In the system, the signal was detected by an EMI 9235QB photomultiplier under a 7.5 mm Hoya U-340 bandpass filter. Optical stimulation was conducted using a dedicated module made of a single high-power LED integrated with an optical adapter inserted into the Risø reader. The LED spectral band was 620 ± 12 nm. The stimulation power used was ~33 mW/cm^2^, and heating was performed in an Ar atmosphere. Irradiation was performed using beta radiation delivered by ^90^Sr/^90^Y β source with a dose rate of 112 mGy s^−1^. The 6 mm diameter mask was used for quartz grains deposition on stainless steel discs delivered by Risø. A silicon spray assured a single-grain layer. The masses of all the used aliquots were comparable (about 5 mg).

### 2.3. Trap Parameter Determination

The values of trap parameters determine the lifetime (*τ*) of charge carriers in a trap, i.e., the mean duration that an electron can be expected to remain trapped. The lifetime for a trap of the depth (or the thermal activation energy) *E* (eV) can be calculated according to the following equation:

(1) $$\tau =s^{-1}\exp\left(\frac{E}{kT}\right),$$

where *s* is the frequency factor (s^−1^) described as the number of escape attempts of an electron from a trap per second [29], *T* is the temperature (K) and *k* is the Boltzmann constant (eV K^−1^).

The OTDC is constructed by the repeated measurement of the remaining OSL signal after keeping the sample at a specific temperature *T* (°C), hereinafter referred to as holding temperature for a different time *t* (from a few to many thousands of seconds), hereinafter referred to as holding time (see Table 1). During such an experiment the thermal release of the electrons from traps related to the OSL is observed. The series of measurements is terminated when the OSL signal is decreased to the PMT background level. One repeats the OTDC measurement for several holding temperatures. Assuming the most basic first-order kinetics and a single kind of depopulated traps, the shape of the OTDC can be described by a simple exponential function (e.g., Refs. [29,30,31]):

(2) $$I\left(t\right)=I_{0}\exp\left(\frac{-t}{\tau }\right),$$

where *I*_0_ is the initial OSL signal for holding time *t* = 0 and *τ* (*s*) is the lifetime of the electron in the trap at temperature *T* (Equation (1)). In this study, the trap parameters (*E* and *s*) have been derived using the TM-OSL_620 nm_ protocol (see Table 1). The lifetime was estimated for each holding temperature by attempting to fit a single exponential function to the OTDC first. The values of *E* and *s* were derived by regression method from the Arrhenius plot (obtained from Equation (1)):

(3) $$\ln\tau =\frac{E}{kT}-\ln s.$$

The plot of ln *τ* versus *1*/*T* yields a straight line of slope *E/k*, and intercept ln *s* on the ordinate axis (e.g., [30]).

#### Protocol

The OTDC protocol used in experiments are shown in Table 1. Samples measured are listed in the legend of Table 1. The OTDC protocol using the TM-OSL_620 nm_ method was described in detail previously [16]. The modification of this protocol applied here consisted in replacing the preheat up to 150 °C with the preheat to the highest holding temperature of those used in the protocol (in this case it was 280 °C). This was conducted in order to remove electrons from shallow traps and to preserve the most similar initial filling of traps for all isothermal decays used in the experiment. The optical stimulation with 620 nm was initiated at 40 °C and continued up to 200 °C whereas linear heating was performed with the rate of 2 Ks^−1^. The heating up to 500 °C was conducted in step 5 to remove all electrons from the traps responsible for the TL signal observed below this temperature. The TM-OSL_620 nm_ signal was taken as the integral under the TM-OSL curve in the range of 40–150 °C. The sum of counts from 21 channels at the maximum of TL peak 110 °C (measurement in step 2 of the protocol in Table 1) was used for correction of the TM-OSL_620 nm_ signal for luminescence sensitivity changes [32]. The OTDCs were normalized to the signal measured for *tj* = 0 s (thus, the y-axis caption is as follows: TM-OSL/TM-OSL (0 s))

In the TM-OSL_620 nm_ protocol, at least two aliquots of each sample were used. One of the aliquots was tested in all the OTDC experiments, and the second aliquot was used to verify the reproducibility of results for selected points of the procedures used.

## 3. Results

### 3.1. Initial Characterization of Luminescence Properties: TL, CW-OSL, TM-OSL

The luminescence properties of the samples were investigated to demonstrate the basic differences between them. TL, CW-OSL_470 nm_ as well as TM-OSL_620 nm_ were detected in the UV detection range (Hoya U-340 filter) after the beta irradiation with a dose of 1000 Gy.

**Figure 1:**
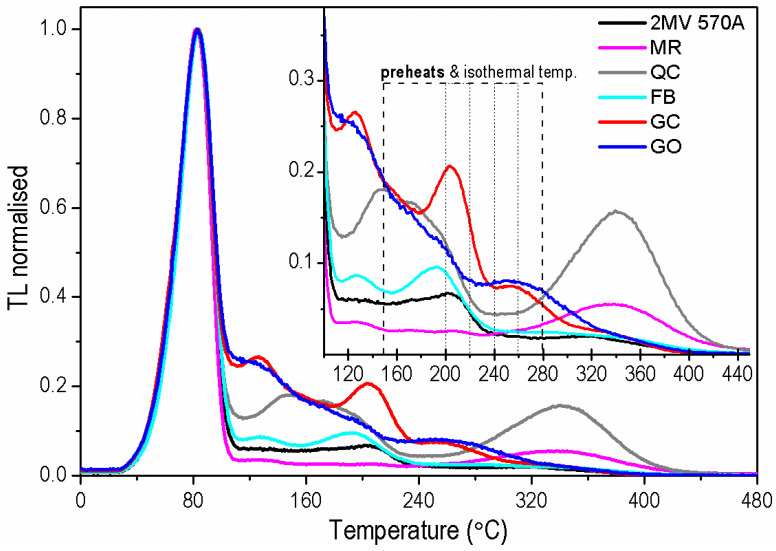
Normalized TL glow curves measured for one aliquot of each sample after irradiation with a dose of 1000 Gy. The inset shows the same data for the high-temperature range with the marked preheat (dashed lines) and storage temperatures (dotted line) used in experiments. TL glow curves were measured immediately after irradiation with heating rate of 2 Ks^−1^ and standard background subtraction was performed for each aliquot.

Figure 1 clearly shows the differences in the shapes of TL curves. Three temperature ranges can be distinguished above 110 °C. In the first one, below 160 °C, all samples except QC have similar shapes of the TL curves. For some of them (GC, FB and MR), the peak at approximately 130 °C is more pronounced, but the overall trend of a slight decrease in TL with temperature is visible. From 160 °C to 240 °C, there is a further general trend of TL decrease, resulting from thermal quenching [33,34,35,36] in which for samples GC, FB and 2 MV 570A, a peak around 200 °C is clearly marked. Between 240 °C and 280 °C, the TL in rock samples QC and GO is distinguished by its stronger intensity (maximum about 260 °C) in contrast to the clear lowering in the signal of other samples. Above 280 °C, the signal of samples MR and QC stands out, with TL curves having a broad TL maximum centered at 340 °C. In the rest of the samples, TL in this range decays slowly, and no particular peaks can be distinguished.

The complex shapes of TL glow curves indicate a multi-trap structure in the investigated quartz samples. The obtained diversity in the TL curve shapes shows that the concentrations of the defects responsible for the individual electron traps in the samples are different.

**Figure 2:**
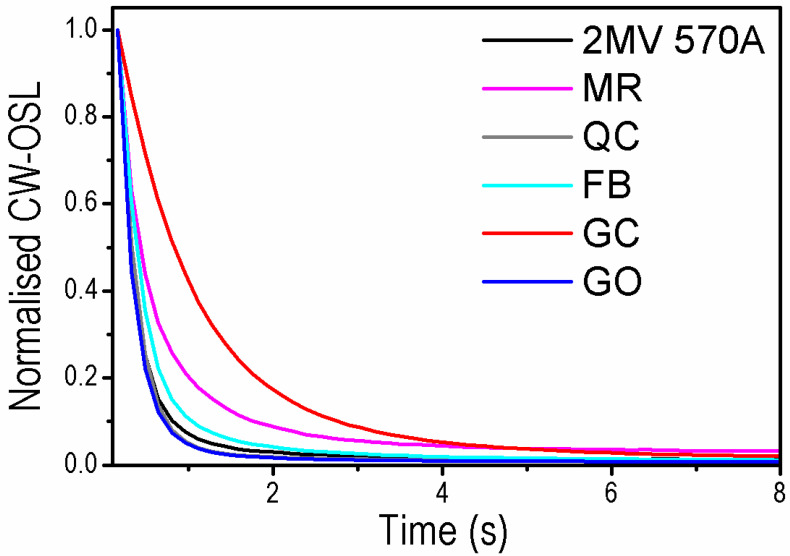
CW-OSL_470 nm_ curves measured for one aliquot of each sample after irradiation with a dose of 1000 Gy and preheat up to 240 °C. Data set after normalization to the initial intensity. The blue LEDs were used for the optical stimulation with 90% of their maximal power ~80 mW/cm^2^.

Based on the CW-OSL_470 nm_ decays (Figure 2) obtained for the same quartz samples, it is possible to distinguish two qualitatively different sample groups with two samples (GC and MR) displaying a slower decay rate than the others. It means that the fast OSL component contribution in the total OSL signal for GC and MR samples is lower than for FB, QC, GO, and 2 MV 570A samples. The observations that the OSL signal originating from FB is dominated by the fast OSL component whereas in the case of the MR sample the total OSL signal is evidently composed of the other OSL components agrees with LM-OSL results for these samples published recently [25]. The LM-OSL curves for both samples were decomposed into several first-order kinetics LM-OSL components. The fast component observed in sample FB was not present in the decomposition results obtained for sample MR.

**Figure 3:**
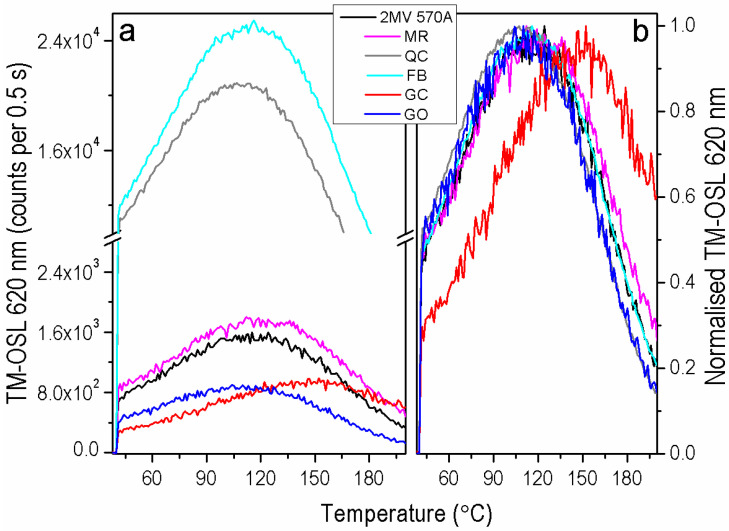
(**a**) TM-OSL_620 nm_ curves measured for one aliquot of each sample after irradiation with a dose of 1000 Gy and preheat up to 280 °C, (**b**) the same data set normalized to the curve maximum. TM-OSL signals were measured after irradiation and preheat up to 280 °C with a heating rate of 2 Ks^−^^1^. During linear heating with a rate of 2 Ks^−^^1^, the optical stimulation was carried out using 620 nm LEDs with power ~33 mW/cm^2^.

The shape of TM-OSL_620 nm_ curves depends on the population of the trap responsible for the fast OSL component. When the fast OSL component is present in the sample, the TM-OSL_620 nm_ curves have a broad-peak shape as presented in Figure 3a, where a comparison of the TM-OSL_620 nm_ curves measured for each of the quartz samples is shown. The brightest in TM-OSL_620 nm_ among the investigated samples are sedimentary quartz FB and QC. Rock quartz samples are less sensitive; however, two sedimentary samples MR and 2 MV 570A are comparable with them in intensities. Differences are visible in the shape of TM-OSL curves for GC and MR samples, where the maximum of the TM-OSL peak is shifted in the higher temperature region; in the case of the MR sample, this shift is slight, whereas for GC is evident (Figure 3b). This behavior agrees with CW-OSL_470 nm_ decays indicating the small contribution of the fast OSL components in these samples. Nevertheless, in the case of these two samples, another OSL component can be observed in the TM-OSL_620 nm_ curve. It manifests itself in a TM-OSL peak at about 150–160 °C, for a heating rate of 2 Ks^−^^1^. In the case of bright samples FB and QC, the predominance of the fast component over the rest of the OSL signal is vast. In this way, the slower component (medium) is not easy for direct observation in the TM-OSL_620 nm_.

### 3.2. TM-OSL Method Used for the Fast OSL Component Separation

**Figure 4:**
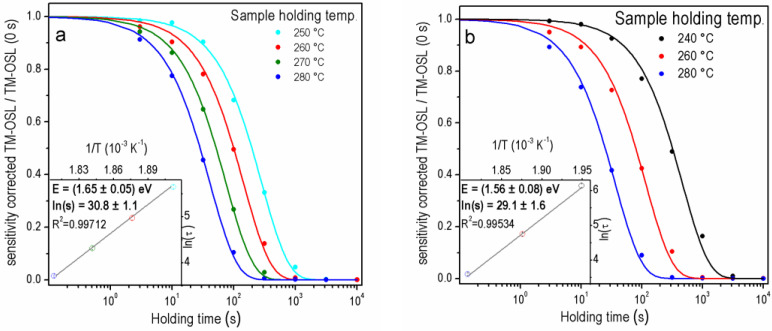
The OTDCs for one aliquot of the FB sample using TM-OSL_620 nm_ procedure after irradiation with dose 1000 Gy and preheating: (**a**) 280 °C, FOM_250_ = 1.89%, FOM_260_ = 3.17%, FOM_270_ = 2.03%, FOM_280_ = 2.33%, (**b**) 150 °C, FOM_240_ = 2.75%, FOM_260_ = 4.13%, FOM_280_ = 3.91%.

As was recently shown [37], the preheat applied immediately after irradiation before starting the storage at a certain holding temperature (step 2 in Table 1) is important not only to remove carriers from shallow traps but also, to ensure a similar level of trap filling for each value of the holding temperature applied in the experiment. In the first stage of the OTDC experiment, the effect of changing the preheat temperature from 150 °C to 280 °C in the TM-OSL_620 nm_ procedure was tested using samples FB and 2 MV 570. The OTDCs obtained for the FB sample (the same aliquot) for the preheat temperatures of 280 °C and 150 °C are shown in Figure 4a and Figure 4b, respectively. For this sample, additional holding temperatures (250 and 270 °C) next to the standard 240, 260, and 280 °C values were applied. Additional holding temperatures were chosen in order to follow thermal depletion processes more carefully. Finally, the best OTDC fits were obtained for holding temperatures: 240, 260, and 280 °C when the preheat temperature of 150 °C was used and for 250, 260, 270 and 280 °C in the case of preheat to 280 °C. When preheat temperature of 150 °C is applied, the experimental points deviate from the exponential curve. The improvement in isothermal decay fitting quality (compare the obtained FOM values in Figure 4a,b) is visible for the preheat to 280 °C. For both samples tested here, when one achieves the better first-order fitting result, i.e., for the higher preheat temperature, the obtained trap depths are higher than for the lower preheat temperature.

**Figure 5:**
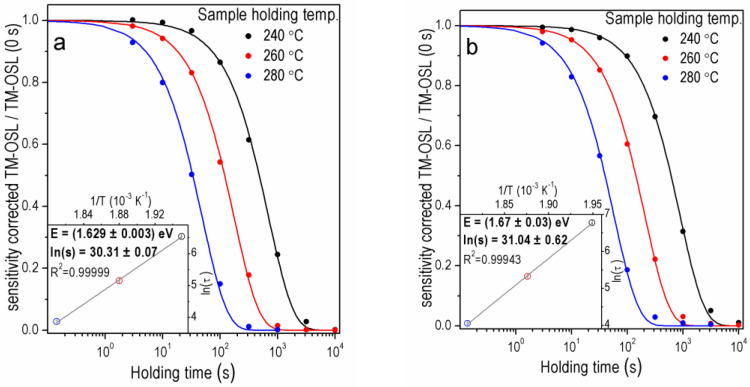
The OTDCs of the quartz samples (**a**) 2 MV 570A for dose 1000 Gy, FOM_240_ = 1.57%, FOM_260_ = 1.53%, FOM_280_ = 2.99%, (**b**) MR for dose 100 Gy, FOM_240_ = 1.17%, FOM_260_ = 1.65%, FOM_280_ = 2.98% for one aliquot and preheating 280 °C.

**Figure 6:**
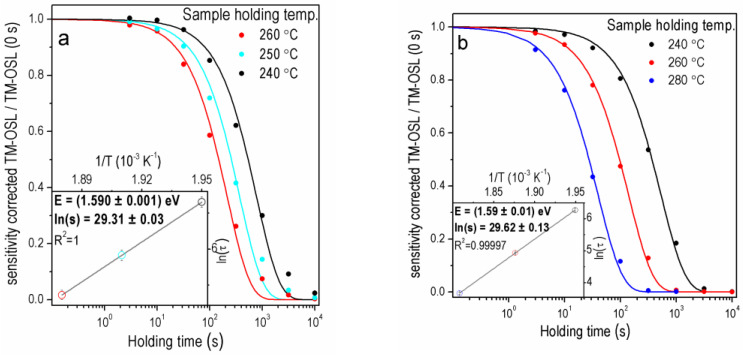
The OTDCs of the granite samples (**a**) Catalina FOM_240_ = 4.46% FOM_250_ = 4.70% FOM_260_ = 5.23% (**b**) Oracle FOM_280_ = 3.57% FOM_260_ = 1.90% FOM_240_ = 2.72% for one aliquot after dose 1000 Gy and preheating 280 °C.

Figure 5 and Figure 6 show, for four samples, the sets of OTDCs created for the OSL signal measured by the TM-OSL_620 nm_ method. The protocol applied, in this case, is shown in Table 1. Results were selected for samples that originate both from sediments and rock and whose contribution of the fast component is different.

Sample 2 MV 570 (Figure 5a) represents the case of sediment quartz with a substantial share of the fast component in the OSL signal. In this case, the first-order curves correctly reproduce the OTDCs. Estimated trap parameters *E* = 1.629 ± 0.003 eV and *s* = (1.46 ± 0.11) × 10^13^ s^−^^1^ have a low value of standard uncertainties. The investigation of OSL components in the sample MR was performed previously [25] and showed that this sample has a very low intensity of the fast OSL component. As shown in Figure 5b, the first-order decay curves can be fitted to OTDCs obtained in these experiments with excellent accuracy. The trap depth and frequency factor estimated for sample MR equal *E* = 1.67 ± 0.03 eV and *s* = (3.0 ± 1.9) × 10^13^ s^−^^1^ are consistent with the values for the rest of the samples.

The TM-OSL method, whose outcomes are shown in Figure 6a,b, enables the construction of OTDCs having regular first-order shapes for both the GO sample with a significant share of the fast component and the GC sample characterized by a low percentage of the fast component in the total OSL signal. It is worth mentioning that the first-order decay fitting for holding a temperature equal to 280 °C for sample granite Catalina is worse than for other temperatures. Therefore, the OTDC for 280 °C was replaced by OTDC measured at 250 °C, for which the first-order fitting gives accurate results. The *E* and *s* (*E* = 1.59 ± 0.01 eV; *s* = (7.3 ± 0.9) × 10^12^ s^−^^1^ for GO and *E* = 1.590 ± 0.001 eV; *s* = (5.3 ± 0.17) × 10^12^ s^−^^1^ for GC) were determined with good precision and agree with those obtained for sediment samples. Summing up, in all the above-presented cases, the first-order decay can be fitted with good quality when the TM-OSL_620 nm_ procedure is applied.

### 3.3. Influence of the Irradiation Dose on the OTDC Measurement Results

**Figure 7:**
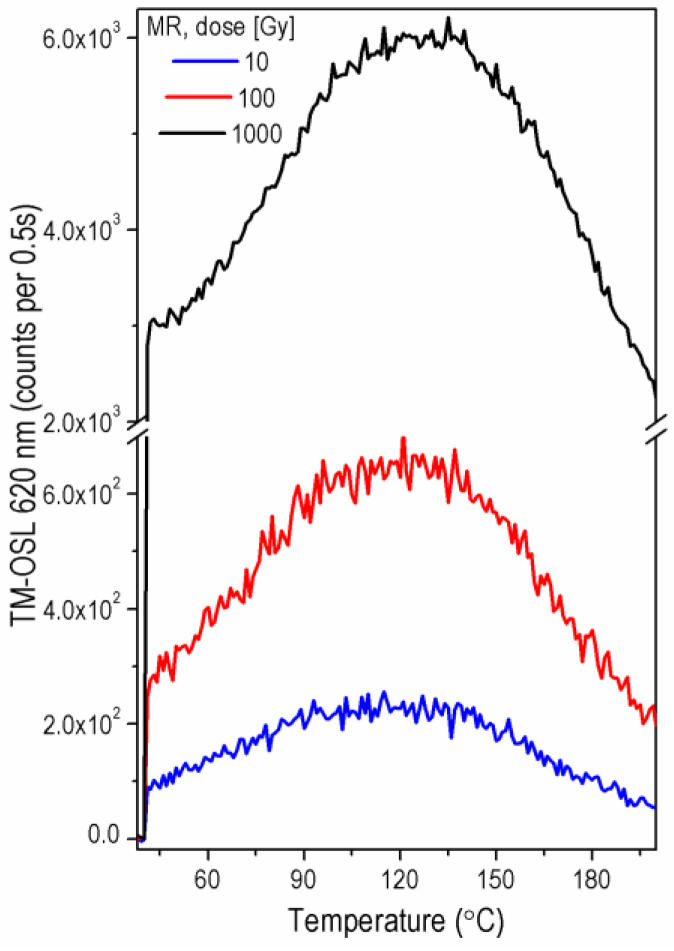
TM-OSL_620 nm_ curves measured for one aliquot of MR sample after irradiation with a dose: 10 Gy (blue), 100 Gy (red) and 1000 Gy (black) and preheat up to 280 °C.

**Figure 8:**
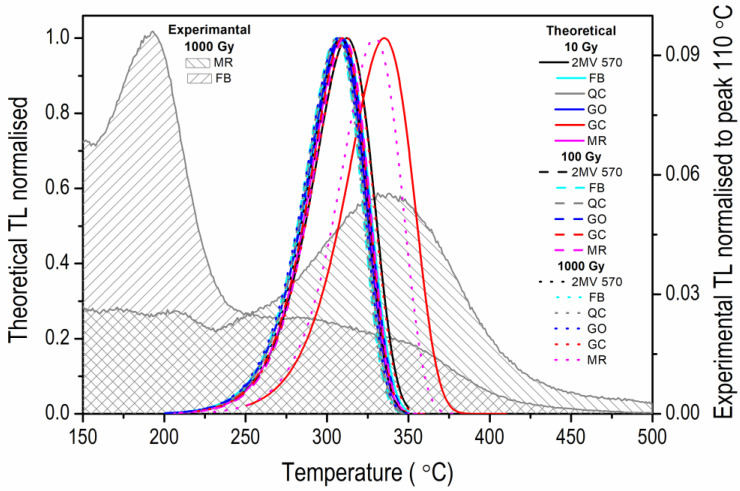
The theoretical single first-order kinetic TL peaks calculated for trap parameters *E* and *s* obtained in OTDC experiments set in Table 2 according to Equation (4) (left vertical axis), heating rate of 2 Ks^−1^. Experimental TL curves (area filled with grey lines) measured using heating rate of 2 Ks^−1^ for MR and FB samples after irradiation with a dose of 1000 Gy and normalisation to peak 110 °C (right vertical axis)—the same data as shown in Figure 1.

The final results of experiments for three different irradiation doses—10 Gy, 100 Gy and 1000 Gy are presented in Table 2, while representative TM-OSL signals for different doses are presented in Figure 7. The first-order kinetics TL peaks are shown in Figure 8 for the *E* and *s* values obtained in these experiments. They are calculated for a heating rate of 2 Ks^−1^. Assuming a linear rate of heating the sample, *b* (Ks^−1^), the TL intensity for the first-order kinetic traps can be expressed as follows [38]:

(4) $$I_{TL}\left(T\right)=sn_{0}\;exp\left(\frac{-E}{kT}\right)exp\left[-\frac{s}{b}\int_{T_{0}}^{T}exp\left(-\frac{E}{kT^{\prime }}\right)dT^{\prime }\right]$$

*n*_0_ (m^−3^) is the number of trapped electrons at the beginning of heating.

When applying the TM-OSL_620 nm_ protocol with a preheat temperature of 280 °C, one can observe some slight variation in parameters with the change of dose. However, no general trend of these changes can be seen. The thermal activation energy varies from 1.54 ± 0.02 eV and 1.54 ± 0.08 eV (the lowest values obtained, respectively, for GC and FB samples, both for dose 10 Gy) up to 1.67 ± 0.31 eV and 1.67 ± 0.03 eV (the highest values obtained for 2 MV 570 and MR samples, both for dose 100 Gy). The frequency factor *s* varies from (5.52 ± 2.33) × 10^11^ s^−1^ (the lowest value obtained for GC when dose 10 Gy was applied) up to (3.14 ± 21.3) × 10^13^ s^−1^ (the highest value obtained for 2 MV 570 when dose 100 Gy was applied). Although the values of frequency factors differ significantly, they are consistent within their uncertainties. The precision of trap parameters also varies from experiment to experiment carried out for one aliquot. The good precision of the majority of *E* results and very high one estimated in a few cases (MR for dose 10 Gy, GC and 2 MV 570 for dose 1000 Gy) contrast with very low precision obtained for samples 2 MV 570 and MR when 100 Gy and 1000 Gy were applied, respectively (Table 2). The low precision of the determined parameters results from the quality of fitting of the exponential curve. Although small, the observed differences in energy values and significant differences in the precision of the parameters determined in OTDC measurements confirm the importance of the dose selection also in the TM-OSL_620 nm_ protocol. Results in Table 2 seem to indicate that the trap parameters obtained for rock samples are slightly lower than those for sediments.

It is worth noting that the positions of the first-order TL peaks obtained for all the results presented in Table 2 (Figure 8) do not differ significantly, except in two cases: sample GC measured with dose 10 Gy (red line) and MR with 1000 Gy (magenta dot line). We attribute this behavior to the dependence of OTDC results on the initial filling of traps active in the processes taking place in the OTDC measurement, as indicated earlier in the simulation study [37] (see Section 4).

## 4. Discussion

Recently, the processes occurring during OTDC measurements in a simple OSL model were studied by simulations using the luminescence kinetics model [37]. The shape of OTDC was presented for a few cases when the measurement for a single OSL trap kind is disturbed by the contribution of other traps in the thermal decay or OSL process. The signal of the investigated OSL traps was assumed to decay according to the first-order kinetics when the process is independent of other traps. These basic investigations allowed us to distinguish two main reasons for OTDCs deformation (1) the optical release of carriers from disturbing traps and their participation in the emission during the OSL measurement, and (2) the thermal transfer of carriers to OSL traps from disturbing traps during the isothermal holding. Both disturbing processes strongly depend on the trap filling at the start of the isothermal holding, so they depend on the irradiation dose and preheat temperature applied in the OTDC protocol.

In light of the research mentioned above, the fundamental problem in OTDC experiments is separating the fast OSL component from other components for investigations. As previously shown, the TM-OSL method with 620 nm wavelength stimulation enables the separation of the fast OSL component in quartz [13,14,15,16]. It is important to note that the method allows for detecting the fast component even in samples in which its intensity is very low. Such a case is shown in Figure 7 for the MR sample for which it was previously shown that the fast component is about 100 times less intense than in well-behaved samples such as sample FB. This component was not detected in sample MR by the LM-OSL measurement with blue light (see [25], (Figure 9) therein).

The OTDC measurement simulations proved that the constant preheat temperature should be used in the whole OTDC protocol and it should be not lower than the highest used holding temperature [37]. This ensures that the filling of traps at the beginning of the isothermal holding is constant in the procedure. However, this is not the only condition that must be met when selecting the correct temperature of the preheat. The preheat should be high enough to empty the shallower traps participating in the OSL and others participating in the thermal transfer during the isothermal holding. This problem, however, also applies to the TM-OSL_620 nm_ protocol, even though the signal from the fast component is well separated. When initially a preheat to 150 °C was used, then the shape of the OTDCs was usually distorted. It is demonstrated in Section 3.2 for samples with the fast OSL component of relatively high intensity. As mentioned, the *E* and *s* values determined with too low preheat temperature are lower than those established with properly selected preheat for protocol TM-OSL_620 nm_ (compare Figure 4a,b) for sample FB. It is worth noting that the reduction in values *E* and *s* means a significant reduction in a trap lifetime. For example, the trap lifetimes in the case of sample 2 MV 570 for the preheat to 280 °C is (2.14 ± 0.04) × 10^5^ ka, for preheat 150 °C it is (1.58 ± 1.70) × 10^4^ ka (calculated for 10 °C).

On the other hand, the selected preheat temperature cannot lead to excessive depopulation of the tested OSL traps. When empty, the shallow traps effectively retrap charge carriers thermally released during the isothermal holding from a deeper trap. Such a pure effect of thermal transfer causes OTDC deformation, especially at longer holding times (see [37], (Figure 13d) therein). In other cases, significant depletion of the tested OSL traps may emphasize in the measured OSL signal the previously negligible contribution from other slower decaying OSL traps in the sample. Then a lowering of trap parameters from OTDC measurement was observed. In the presented measurement results, such a situation seems to occur for sample GC with a low intensity of the fast OSL component. The OTDC experiment carried out using the TM-OSL protocol with the irradiation dose of 10 Gy led to the *E* and *s* values of 1.54 ± 0.02 eV and (5.52 ± 2.33) × 10^11^ s^−1^, respectively. These values are lower than those obtained for the same aliquot of this sample but for higher doses (Table 2). The first-order peak for these parameters is shifted into high temperatures from the peak obtained for other doses in the case of sample GC and the rest of the samples by more than 20 °C.

The above example indicates the significance of the irradiation dose selection in the OTDC protocol. This has been demonstrated by the simulation study mentioned above. When a preheat temperature that ensures that the shallower traps are emptied is applied before isothermal holding, it may turn out that the initial occupation of the investigated OSL traps is very low. This can lead to a low signal-to-background ratio. Then, using a higher irradiation dose in the protocol helps obtain better precision in the OSL measurements. However, the higher dose used in the measurements does not always guarantee the correct determination of the parameters of the tested OSL traps. As shown, the crucial condition is the significant dominance of the signal from these traps in the total OSL signal measured in the OTDC protocol. Applying a higher dose does not necessarily make the condition true. It is the case, for example, when other slower decaying, thermally deeper OSL traps have a significantly higher concentration and, due to a lower retrapping factor, they fill up more slowly. For the doses for which the tested OSL traps are full, the share of the total OSL signal of the slower traps begins to play an increasingly important role. Hence, when a high dose that is too high is applied, the stipulation of the tested trap dominance in the total OSL signal is weakened and the resulting OTDCs have a slower decay.

In Figure 8, one can notice another example of the TL peak shifting from the others to higher temperatures. It is the curve calculated for the *E* and *s* obtained for sample MR, hence again for a sample with a relatively low intensity of the fast OSL component. The case corresponds to the above-mentioned situation when a too-high dose is used in the OTDC protocol. One can fit the OTDCs by an exponential decay with reasonable accuracy, but the frequency factor assuring this result is much lower than for smaller doses (*s* = (4.51 ± 14.4) × 10^12^ s^−1^ versus *s* = (2.28 ± 0.19) × 10^13^ s^−1^ for doses 1000 Gy and 10 Gy, respectively). Its low value determines the position of the calculated TL peak on the temperature axis. In the case of other samples, such a high dose did not cause a similarly severe parameter deviation, indicating that the effects are sample-dependent. Intuitively, they should be such when one explains deformations of OTDCs by processes resulting from the competition of various traps. The latter is controlled by trap concentrations, which may vary significantly from sample to sample and, in the case of sediments, from grain to grain. It means that one should not arbitrarily apply a once-selected OTDC procedure to all tested samples but check how the results change with the preheat temperature and dose. The shape of OTDCs and the quality of curve fitting should be carefully viewed and selected for trap parameter estimation.

## 5. Conclusions

Trap parameters estimated by OTDC measurements may depend on various measurement parameters applied. The significant discrepancy in the values of the trap depth and the frequency factor of traps responsible for the fast OSL component in quartz previously presented in the literature is most probably a consequence of the complexity of the processes occurring in quartz during OTDC measurements. It was demonstrated that a careful selection of the measurement parameters and the protocols used is needed. To sum up:

- Both in samples with a low share and with a dominant share of the fast component in the total OSL signal, the proper separation of the fast OSL component in the OSL measurement is crucial for obtaining reliable values of trap parameters.
- A procedure using the TM-OSL method for measurement of the isothermal depletion curve with preheat 280 °C seems to be the best solution, at least in the case of the investigated samples. This is supported by the fact that a single exponential function is sufficient to obtain a good reconstruction of OTDC even for quartz samples of low OSL fast component intensity.
- For samples not dominated by the fast OSL component, the OTDCs can be more stretched and the fit quality can decrease. However, if the TM-OSL signal is measurable, probably after finding the proper dose, preheat and thermal holding temperatures, the trap stability may be successfully designated still using the first-order kinetic decay.
- The trap responsible for the fast OSL component in quartz is independent of the quartz sample type (sediment or rock). The mean trap depth and *s* factor (calculated based on bold data from Table 2) are: *E* = 1.63 ± 0.01 eV, *s* = (1.83 ± 0.37) × 10^13^ s^−1^ and are close to these established earlier for sedimentary quartz that had the fast component dominating in OSL signal by Murray and Wintle [20]: *E* = 1.66 ± 0.03 eV, *s* = (1.00 ± 1.99) × 10^13^ s^−1^.

Overall, we showed that the TM-OSL_620 nm_ method used in the isothermal procedure can be used independently of quartz origin even for low sensitivity samples if the TM-OSL signal is measurable and experiments are carried out for proper values of dose, preheat, and isothermal holding temperatures.

## Figures and Tables

**Table 1:** OTDC protocol used in the experiments.

	Protocol TM-OSL_620 nm_
**1**	Irradiation (D) *
**2**	Preheat to **150 °C or 280 °C**, 2 Ks^−1^, 0 s
**3**	Heating the sample to T ** with the heating rate of 1 Ks^−1^ and holding at T during t *** seconds
**4**	TM-OSL_620 nm_ from 40 °C to 200 °C, 2 Ks^−1^
**5**	Heating to 500 °C, 5 Ks^−1^
**6**	Go to step 1

Sample measured: 2 MV 570A, GO, GC; QC, FB, MR; *—10 Gy, 100 Gy, 1000 Gy; **—sample holding temperatures in °C: 240, 250, 260, 270, 280; ***—sample holding times in seconds: 0, 3, 10, 32, 100, 316, 1000, 3160, 10,000.

**Table 2:** Trap parameters values (*E* and *s*) obtained for various irradiation doses in OTDC experiment using TM-OSL_620 nm_ protocol for preheat 280 °C. T_max_ values are the maxima of TL peak positions simulated using first-order kinetic for adequate *E*, *s* and heating rate of 2 K/s.

		Rocks		Sediments			
		GO	GC	2 MV 570	FB	QC	MR
**10** **[Gy]**	E [eV]	1.60 ± 0.11	1.54 ± 0.02	1.56 ± 0.04	1.54 ± 0.08	1.64 ± 0.05	**1.653 ± 0.004**
	s [s^−1^]	(8.3 ± 19.0) × 10^12^	(5.52 ± 2.33) × 10^11^	(2.96 ± 2.51) × 10^12^	(2.21 ± 3.88) × 10^12^	(1.98 ± 2.02) × 10^13^	**(2.28 ± 0.19) × 10^13^**
	T_max_ [°C]	307.7	335.3	312.1	309.3	307.3	309.3
**100** **[Gy]**	E [eV]	**1.56 ± 0.03**	**1.63 ± 0.03**	1.67 ± 0.31	**1.64 ± 0.03**	**1.65 ± 0.01**	**1.67 ± 0.03**
	s [s^−1^]	**(3.43 ± 2.15) × 10^12^**	**(1.4 ± 0.9) × 10^13^**	(3.14 ± 21.3) × 10^13^	**(2.15 ± 1.38) × 10^13^**	**(2.50 ± 0.32) × 10^13^**	**(3.0 ± 1.9) × 10^13^**
	T_max_ [°C]	308.9	309.4	310.0	305.8	306.4	310.6
**1000** **[Gy]**	E [eV]	**1.59 ± 0.01**	1.590 ± 0.001	**1.629 ± 0.003**	1.65 ± 0.05	**1.65 ± 0.04**	1.63 ± 0.15
	s [s^−1^]	**(7.3 ± 0.9) × 10^12^**	(5.3 ± 0.17) × 10^12^	**(1.46 ± 0.11) × 10^13^**	(2.47 ± 2.72) × 10^13^	**(2.60 ± 2.14) × 10^13^**	(4.51 ± 14.4) × 10^12^
	T_max_ [°C]	306.5	312.4	308.5	306.9	306.1	329.0

## Data Availability

All data was made open access on Zenodo and is digitally identified by DOI https://doi.org/10.5281/zenodo.7383041.

## References

[1] Rhodes E.J. (2011). Optically stimulated luminescence dating of sediments over the past 200,000 years. Annu. Rev. Earth Planet Sci..

[2] Guralnik B., Jain M., Herman F., Paris R.B., Harrison T.M., Murray A.S., Valla P.G., Rhodes E.J. (2013). Effective closure temperature in leaky and/or saturating thermochronometers. Earth Planet. Sci. Lett..

[3] Smith B.W., Rhodes E.J. (1994). Charge movements in quartz and their relevance to optical dating. Radiat. Meas..

[4] Bailey R.M., Smith B.W., Rhodes E.J. (1997). Partial bleaching and the decay form characteristics of quartz OSL. Radiat. Meas..

[5] Murray A.S., Wintle A.G. (2003). The single aliquot regenerative dose protocol: Potential for improvements in reliability. Radiat. Meas..

[6] Li B., Li S.H. (2006). Comparison of De estimates using the fast component and the medium component of quartz OSL. Radiat. Meas..

[7] Wang X.L., Wintle A.G., Lu Y.C. (2006). Thermally transferred luminescence in fine-grained quartz from Chinese loess: Basic observations. Radiat. Meas..

[8] Wu T.S., Jain M., Guralnik B., Murray A.S., Chen Y.G. (2015). Luminescence characteristics of quartz from Hsuehshan Range (Central Taiwan) and implications for thermochronometry. Radiat. Meas..

[9] Durcan J.A. (2018). Assessing the reproducibility of quartz OSL lifetime determinations derived using isothermal decay. Radiat. Meas..

[10] Mineli T.D., Sawakuchi A.O., Guralnik B., Lambert R., Jain M., Pupim F.N., del Rio I., Guedes C.C.F., Nogueira L. (2021). Variation of luminescence sensitivity, characteristic dose and trap parameters of quartz from rocks and sediments. Radiat. Meas..

[11] King G.E., Herman F., Lambert R., Valla P.G., Guralnik B. (2016). Multi-OSL-thermochronometry of feldspar. Quat. Geochronol..

[12] Faershtein G., Guralnik B., Lambert R., Matmon A., Porat N. (2018). Investigating the thermal stability of TT-OSL main source trap. Radiat. Meas..

[13] Chruścińska A., Kijek N. (2016). Thermally modulated optically stimulated luminescence (TM–OSL) as a tool of trap parameter analysis. J. Lumin..

[14] Chruścińska A., Kijek N., Topolewski S. (2017). Recent development in the optical stimulation of luminescence. Radiat. Meas..

[15] Chruścińska A., Szramowski A. (2018). Thermally modulated optically stimulated luminescence (TM-OSL) of quartz. J. Lumin..

[16] Biernacka M., Chruścińska A., Palczewski P., Derkowski P. (2022). Determining the kinetic parameters of traps in quartz using the thermally modulated OSL method. J. Lumin..

[17] Dodson M.H. (1973). Closure temperature in cooling geochronological and petrological systems. Contrib. Mineral. Petrol..

[18] Herman F., Rhodes E.J., Braun J., Heiniger L. (2010). Uniform erosion rates and relief amplitude during glacial cycles in the Southern Alps of New Zealand, as revealed from OSL-thermochronology. Earth Planet. Sci. Lett..

[19] Li B., Li S.H. (2012). Determining the cooling age using luminescence-thermochronology. Tectonophysics.

[20] Murray A.S., Wintle A.G. (1999). Isothermal decay of optically stimulated luminescence in quartz. Radiat. Meas..

[21] Spooner N.A., Questiaux D.G. (2000). Kinetics of red, blue and UV thermoluminescence and optically-stimulated luminescence from quartz. Radiat. Meas..

[22] Groza-Săcaciu Ș.M., Panaiotu C., Timar-Gabor A. (2020). Single Aliquot Regeneration (SAR) Optically Stimulated Luminescence Dating Protocols Using Different Grain-Sizes of Quartz: Revisiting the Chronology of Mircea Vodă Loess-Paleosol Master Section (Romania). Methods Protoc..

[23] Madsen A.T., Murray A.S., Andersen T.J. (2007). Optical dating of dune ridges on Rømø, a barrier island in the Wadden Sea, Denmark. J. Coast. Res..

[24] Hansen V., Murray A., Buylaert J.P., Yeo E.Y., Thomsen K. (2015). A new irradiated quartz for beta source calibration. Radiat. Meas..

[25] Schmidt C., Chruścińska A., Fasoli M., Biernacka M., Kreutzer S., Polymeris G.S., Sanderson D.C.W., Cresswell A., Adamiec A., Martini M. (2022). A systematic multi-technique comparison of luminescence characteristics of two reference quartz samples. J. Lumin..

[26] Kreutzer S., Friedrich J., Sanderson D., Adamiec G., Chruścińska A., Fasoli M., Martini M., Polymeris G.S., Burbidge C.I., Schmidt C. (2017). Les sables de fontainebleau: A natural quartz reference sample and its characterization. Anc. TL.

[27] Fornash K.F., Patchett P.J., Gehrels G.E., Spencer J.E. (2013). Evolution of granitoids in the Catalina metamorphic core complex, southeastern Arizona: U–Pb, Nd, and Hf isotopic constraints. Contrib. Mineral. Petrol..

[28] Ducea M.N., Triantafyllou A., Krcmaric J. (2020). New timing and depth constraints for the Catalina metamorphic core complex, southeast Arizona. Tectonics.

[29] Aitken M.J. (1985). Thermoluminescence Dating.

[30] McKeever S.W.S. (1985). Thermoluminescence of Solids.

[31] Chen R., McKeever S.W.S. (1997). Theory of Thermoluminescence and Related Phenomena.

[32] Wintle A.G., Murray A.S. (2006). A review of quartz optically stimulated luminescence characteristics and their relevance in single-aliquot regeneration dating protocols. Radiat. Meas..

[33] Wintle A.G. (1975). Thermal quenching of thermoluminescence in quartz. Geophys. J. R. Astron. Soc..

[34] Subedi B., Oniya E., Polymeris G.S., Afouxenidis D., Tsirliganis N.C., Kitis G. (2011). Thermal quenching of thermoluminescence in quartz samples of various origin. Nucl. Instrum. Methods Phys. Res. B.

[35] Subedi B., Polymeris G.S., Tsirliganis N.C., Pagonis V., Kitis G. (2012). Reconstruction of thermally quenched glow curves in quartz. Radiat. Meas..

[36] Friedrich J., Kreutzer S., Schmidt C. (2018). Radiofluorescence as a detection tool for quartz luminescence quenching processes. Radiat. Meas..

[37] Pawlak N.K., Chruścińska A., Biernacka M., Palczewski P. (2022). Thermal stability assessment of OSL signal by measuring the OSL thermal depletion curves. Measurement.

[38] Randall J.T., Wilkins M.H.F. (1945). Phosphorescence and electron traps. I. The study of trap distributions. Proc. R. Soc. Lond. Ser. A.

